# Acid‐sensing ion channels regulate nucleus pulposus cell inflammation and pyroptosis via the NLRP3 inflammasome in intervertebral disc degeneration

**DOI:** 10.1111/cpr.12941

**Published:** 2020-10-27

**Authors:** Kangcheng Zhao, Ran An, Qian Xiang, Gaocai Li, Kun Wang, Yu Song, Zhiwei Liao, Shuai Li, Wenbin Hua, Xiaobo Feng, Xinghuo Wu, Yukun Zhang, Abhirup Das, Cao Yang

**Affiliations:** ^1^ Department of Orthopaedics Union Hospital Tongji Medical College Huazhong University of Science and Technology Wuhan 430022 China; ^2^ Department of Plastic Surgery Union Hospital Tongji Medical College Huazhong University of Science and Technology Wuhan 430022 China; ^3^ SpineLabs St George & Sutherland Clinical School The University of New South Wales Sydney NSW Australia; ^4^ Spine Service Department of Orthopaedic Surgery St George Hospital Campus Sydney NSW Australia

**Keywords:** acid‐sensitive ion channel, intervertebral disc degeneration, NLRP3 inflammasome, pyroptosis

## Abstract

**Objective:**

Lactate accumulation is an important factor in the intervertebral disc degeneration (IVDD). Currently, the effect and underlying mechanism of action of lactate on nucleus pulposus (NP) cell inflammation during IVDD are unclear. Previous studies have found that the NLRP3 inflammasome plays an important role in the regulation of NP inflammation. This study focused on the regulation of acid‐sensitive ion channels (ASICs) in relation to inflammation and the effect of NLRP3 on pyroptosis levels in NP cells under acidic conditions.

**Design:**

For the in vitro experiments, human NP cells were exposed to 6 mM lactate solution; different groups were either treated with NLRP3 inhibitor or transfected with siRNA against NLRP3, siRNA against ASC or a mix of these, and mRNA and protein expression levels were then assessed. For the in vivo experiment, varying concentrations of lactate were injected into rat intervertebral discs and examined via magnetic resonance imaging (MRI) and histological staining.

**Results:**

Extracellular lactate promoted NLRP3 inflammasome activation and degeneration of the NP extracellular matrix; furthermore, it increased the levels of inflammation and pyroptosis in the NP. Lactate‐induced NLRP3 inflammasome activation was blocked by ASIC inhibitors and NLRP3 siRNA.

**Conclusions:**

Extracellular lactate regulates levels of intercellular reactive oxygen species (ROS) through ASIC1 and ASIC3. ROS activate the NF‐κB signalling pathway, thus promoting NLRP3 inflammasome activation and IL‐1β release, both of which promote NP degeneration.

## Graphical abstract

Schematic Illustration of NLRP3 Inflammasome Activation and Pyroptosis in Lactate‐stimulated Human NP Cells. Extracellular lactate induces Ca^2+^ influx by stimulating ASIC1a and ASIC3 on the cell membrane. As a second messenger, Ca^2+^ promotes an increase in intracellular ROS. ROS increases NLRP3 inflammasome component expression through the NF‐κB signaling pathway. NLRP3 components assemble and activate in the cells, leading to IL‐1β release and pyroptosis.

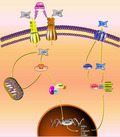

## 1 INTRODUCTION

Low back pain (LBP) is becoming increasingly prevalent amid an ageing society, with up to 80% of adult of all ages having experienced it at some time. Although non‐lethal, LBP can limit activity levels and cause a heavy socio‐economic burden.^1^ Intervertebral disc (IVD) degeneration (IVDD) is the most frequent cause of LBP; however, the molecular mechanisms of IVDD are unclear. Because of this, effective therapies that can be applied to back pain, including growth factor injection, cell transplantation and gene therapy, are not effective for LBP.

Intervertebral discs lie between adjacent vertebral bodies and maintain the stability of the spine by providing flexibility and load support. IVDs are composed of three discrete components: the nucleus pulposus (NP), the annulus fibrosus (AF) and the cartilage endplate (CEP). The hydrogel‐like NP tissues are surrounded by the peripheral AF and lie between adjacent CEPs. The gelatinous NP is the main functional component that enables the discs to withstand diverse mechanical impacts.^2^ NP cells are the primary cell type comprising the NP. Extracellular matrix (ECM) molecules such as collagen II and proteoglycan are generated by NP cells and constitute the main components of the NP.^3^ Dysfunction of NP tissues is known to play an important role in the pathological processes of IVDD.^4^, ^5^ Therefore, increasing the number of functional NP cells is considered to be a key to rebuilding the function of IVDs.

Recent studies have reported that inflammation plays a crucial role in the pathological processes of IVDD, such as matrix degradation, cell senescence and apoptosis.^6^, ^7^ A common hallmark of IVDD is an increase in the levels of inflammatory cytokines, such as tumour necrosis factor‐α (TNF‐α) and interleukin‐1β (IL‐1β). These inflammatory cytokines can disrupt the balance of ECM degradation and synthesis^8^ and promote cell death.^9^ Analyses of both healthy and degenerated discs have revealed that the level of the inflammatory cytokine IL‐1β is elevated in the latter.

As the NP tissues located at the centre of IVDs are avascular,^10^ nutrient and metabolite transport are dependent on permeation through endplates. Due to low oxygen tension in IVDs,^11^ anaerobic glycolysis provides the main source of energy in NP cells.^12^ Conditions such as ischaemia, inflammation and hypoxia give rise to tissue acidosis. A previous study demonstrated that the lactate concentration is higher in the degenerative disc tissues than that in the blood and other tissues.^13^, ^14^ An investigation into the lactate concentrations in LBP patients showed that these largely varied from 2 to 6 mM in the inner annulus.^13^ Although lactate is known to be an important factor in promoting IVDD and regulating NP cell apoptosis, autophagy, matrix synthesis and inflammation,^14^, ^15^ the underlying mechanism by which lactate affects NP cells during IVDD is unclear.

The acid‐sensing ion channels (ASICs) represent a subfamily of proton‐gated channels that comprise six isoforms (ASIC1a, 1b, 2a, 2b, 3 and 4) and are activated by extracellular acidosis, lactate and arachidonic acid.^16^ ASICs are widely distributed throughout various mammalian tissues, such as the nervous system,^17^, ^18^ articular chondrocytes,^19^ and musculoskeletal and IVD cells.^20^, ^21^ These channels are composed of three protein subunits, which may be identical or different,^22^, ^23^ and are involved in Na^+^, K^+^ and Ca^2+^ flux.^24^, ^25^ ASICs have been shown to play crucial roles in modulating cell physiological and pathological processes, such as differentiation,^26^ apoptosis^19^ and autophagy.^27^ ASICs are implicated in the processes of IVDD, which is characterized by a reduction in NP cells and the destruction of the ECM. A recent study demonstrated that ASIC1, ASIC2 and ASIC3 are significantly increased in degenerative NP cells.^28^ More importantly, some studies have reported that ASIC1a regulates endplate chondrocyte apoptosis via Ca^2+^ and increases matrix metalloproteinase activity through the nuclear factor‐κB (NF‐κB) signalling pathway in endplate chondrocytes.^29^, ^30^

Inflammasomes are cytosolic multiprotein complexes that play important roles in infectious, autoimmune and metabolic diseases.^31^ Thus far, five different inflammasomes have been identified, including NLRP1, NLPR3, NLRC4, pyrin and absent in melanoma 2 (AIM2). NLRP3 is the most studied inflammasome. The inflammasome component consists of three members: the NOD‐like receptor, cysteine protease caspase‐1 and the adaptor protein apoptosis‐associated speck‐like protein containing a CARD (ASC). NLRP3 inflammasomes can be activated by various factors, including pathogen‐associated molecular patterns (PAMPs), such as lipopolysaccharide (LPS); microorganisms; viruses; and damage‐associated molecular patterns (DAMPs) such as extracellular ATP, uric acid and cholesterol crystals. Until now, a two‐step model of NLRP3 activation has been documented, involving priming and activation. In the first step, NLRP3 needs to be ‘primed’ by toll‐like receptor agonists; this step not only transcriptionally upregulates NLRP3 expression but also post‐transcriptionally activates NLRP3 by phosphorylation and deubiquitination. In the second step, NLRP3 recruits ASC and pro‐caspase‐1 for NLRP3 inflammasome oligomerization, leading to IL‐1β release and cell pyroptosis. There is accumulating evidence of NLRP3‐mediated inflammation in various highly prevalent diseases, such as diabetes, neurodegeneration and cardiovascular diseases. IL‐1β has been shown to be highly expressed in degenerated human IVDs and to play a critical role in the pathogenesis of IVDD.^32^ In addition, it has been demonstrated that the expression level of NLRP3 and its downstream targets, caspase‐1 and IL‐1β, are positively correlated with Pfirrmann IVDD grade.^33^

The above findings suggest that ASICs play important roles in the inflammatory processes associated with IVDD. However, it is unclear whether ASICs regulate inflammation in NP cells, and if so, by what mechanism. We therefore hypothesized that ASIC1a and ASIC3 contribute to the inflammation of NP cells that are stimulated by extracellular lactate. In this study, we stimulated normal IVDs in vitro and in vivo with lactate, a common extracellular acid, to reveal the relationship between ASICs and NLRP3 inflammasome activation.

## 2 METHODS

### 2.1 Clinical tissue samples

Experimental protocols were approved by the Ethics Committee of Tongji Medical College, Huazhong University of Science and Technology. Degenerative NP tissues were collected from patients undergoing surgery due to IVDD (male, 3; female, 4; age, 35‐68 years, mean = 56.4 years; Pfirrmann level ≥ IV). Healthy tissues were collected from patients with mild IVDD (male, 4; female, 3; age, 12‐20 years, mean = 16.5 years; Pfirrmann level ≤ II), who underwent surgery for idiopathic scoliosis or vertebral fracture. Specimens were immediately sectioned for use in various experiments. One section was fixed in 4% buffered formaldehyde (pH 7.4) for histological analysis. A second section was immersed in the RNAlater solution for use in protein and RNA analysis. Another section was immersed in phosphate‐buffered saline (PBS) for cell isolation.

### 2.2 Isolation and culture of human NP cells

Seven human NP tissues were obtained from patients displaying mild degeneration; these were used to isolate NP cells, which were then plated and expanded for 3 weeks at 37°C and 5% CO_2_ in Dulbecco's modified Eagle medium containing 15% foetal bovine serum (Gibco) and 1% penicillin/streptomycin (Invitrogen). The culture medium was replaced twice per week. Cells from the second passage were used in further experiments. Fluorescent‐activated cell sorting was used to identify NP cell markers (CD24, 311117; KRT18, 628404; Biolegend) with cells from the second passage. NP cells were exposed to 6 mM lactate solution for 24 hours for the in vitro experiments. For NLRP3 inflammasome inhibition, NP cells were treated with MCC950 (10 μM). For *NLRP3* and *ASC* knockdown, NP cells were transfected for 48 hours with 100 nM siRNA against NLRP3, 100 nM siRNA against ASC or 100 nM mixed siRNA (GeneChem) using Lipofectamine 2000 (Invitrogen).

### 2.3 Reverse transcription and quantitative real‐time PCR

Total RNA was extracted with TRIzol reagent (Aidlab) from NP tissues and cultured cells and then reverse transcribed according to the manufacturer's instructions. Briefly, 1 μL total RNA was reverse transcribed using an All‐in‐One First‐Strand cDNA Synthesis Kit (GeneCopoeia), and 1 μL of the resulting cDNA was amplified through PCR with an ABI7900 Eco Real‐Time PCR system (Illumina) using 200 μM specific primers and 10 μL 2X SYBR Green/Fluorescein qPCR Master Mix. PCR tubes were incubated at 50°C for 2 min and 95°C for 10 minutes, followed by 40 cycles at 95°C for 30 seconds and 60°C for 30 seconds. Results were normalized against *GAPDH* according to the $2^{‐\Delta \Delta C_{t}}$ method. The primer sequences used for qRT‐PCR are listed in Table 1.

**TABLE 1:** Sequences of primers used for RT‐PCR

Gene	Primer	Sequence
NLRP3	Forward	5′‐TTCGGAGATTGTGGTTGGG‐3′
	Reverse	5′‐AGGGCGTTGTCACTCAGGT‐3′
ASC	Forward	5′‐AGCTCCCCTACCTTCCACAT‐3′
	Reverse	5′‐AAATCTGCTTTGGTGGTTGG‐3′
Caspase‐1	Forward	5′‐AAAGGGATGAAAGGTGTGCTT‐3′
	Reverse	5′‐CCAAGAATGTGCTGTCTGAGTT‐3′
IL‐1β	Forward	5′‐ATGGCTTATTACAGTGGCA‐3′
	Reverse	5′‐TGTAGTGGTGGTCGGAGA‐3′
MMP3	Forward	5′‐GATGCGCAAGCCCAGGTGTG‐3′
	Reverse	5′‐GCCAATTTCATGAGCAGCAACGA‐3′
MMP13	Forward	5′‐TCAGGAAACCAGGTCTGGAG‐3′
	Reverse	5′‐TGACGCGAACAATACGGTTA‐3′
ADAMT4	Forward	5′‐ACCCAAGCATCCGCAATC‐3′
	Reverse	5′‐TGCCCACATCAGCCATAC‐3′
ADAMT5	Forward	5′‐GACAGTTCAAAGCCAAAGACC‐3′
	Reverse	5′‐TTTCCTTCGTGGCAGAGT‐3′
CollagenⅡ	Forward	5′‐AATTCCGACCTCGTCATCAG‐3′
	Reverse	5′‐GCCTGGATAACCTCTGTG‐3′
aggrecan	Forward	5′‐TGAGCGGCAGCACTTTGAC‐3′
	Reverse	5′‐TGAGTACAGGAGGCTTGAGG‐3′
GAPDH	Forward	5′‐GCCGCTTCTTCTCGTGCAG‐3′
	Reverse	5′‐ATGGATCATTGATGGCGACAACAT‐3′

### 2.4 Western blotting

Tissue or cellular protein was lysed and extracted with cold lysis buffer (Beyotime) for 20 minutes. The proteins were separated via 10% sodium dodecyl sulphate polyacrylamide gel electrophoresis and transferred to a polyvinylidene difluoride membrane that was blocked with Tris‐buffered saline with 0.1% non‐fat milk for 2 hours at room temperature; the membrane was then incubated overnight at 4°C with antibodies. Primary antibody dilution rates varied from 1:500 to 1:1000 for collagen II (Abcam, ab34712), aggrecan (Abcam, ab3778), MMP3 (Abcam, ab53015), MMP13 (Abcam, ab39012), ADAMT4 (Abcam, ab84792), ADAMT5 (Abcam, ab41037), ASIC1a (Abcam, ab240896), ASIC3 (Abcam, ab49333), GSDMD‐N (Abcam, ab215203), NLRP3 (Abcam, ab214185), ASC (Abcam, ab180799), caspase‐1 (Santa, sc‐3071), IL‐1β (Abcam, ab2105), phosphorylated IκBα (CST, #9242) and phosphorylated p65 (CST, #3033). After five washes with TBST, the membrane was incubated with a horseradish peroxidase‐conjugated secondary goat anti‐rabbit IgG antibody for 2 hours at 37°C. GAPDH was used as the protein loading control.

### 2.5 ELISA and LDH release assay

Cell culture supernatants were collected and stored at −80°C until use. ELISA kits detecting human IL‐1β were obtained from R&D systems. Assays were performed according to the manufacturer's instructions using a sample volume of 100 μL. Absorbances at 450 or 490 nm in the LDH release assays, with a correction wavelength of 610 nm, were detected on a SpectraMax M2 fluorescence microplate reader using SoftMax Pro version 5 Software (Molecular Devices). Measurements were performed in duplicate.

### 2.6 Flow cytometry

To assess pyroptosis in human NP cells, active caspase‐1 was determined using the FLICA 660 in vitro Caspase‐1 Detection kit (FLICA 660‐YVAD‐FMK) as described previously. SYTOX Green stain, a fluorescent nucleic acid dye that only penetrates ruptured cell membranes, was used to mark cells with membrane pore formation. The cells were harvested and incubated with a red caspase‐1 detection probe (FLICA 660‐YVAD‐FMK) for 60 minutes at 37°C in the dark. At the end of incubation, the unbound FLICA reagent was washed away using cell wash buffer. Cells were then stained with 1 μM SYTOX Green for 10 minutes at 37°C in darkness. The cells were then analysed using a flow cytometer (BD Biosciences, San Jose, CA, USA). Pyroptotic cells were defined as double positive for FLICA 660‐YVAD‐FMK and SYTOX Green.

### 2.7 Propidium iodide (PI) staining

To confirm pore formation in the cell membrane, PI staining was performed after the treatments. The cells were seeded in a 24‐well plate and allowed to adhere for 24 hours. Following treatment as mentioned above, the cells were washed with PBS and then stained with Hoechst 33342 (5 mL) and PI (5 mL) for 20 minutes at 37°C in darkness. Cells with blue and red fluorescence were observed using a fluorescence microscope (Leica). Approximately 200 cells were identified as PI‐positive cells out of three random microscopic fields for each sample; these were expressed as the percentage of the total cell number.

### 2.8 Immunofluorescence analysis

NP cells were harvested, washed three times with PBS and fixed with 4% formaldehyde for 15 minutes at room temperature. Cells were incubated overnight at 4°C with primary antibodies (1:200) against collagen II and labelled for 40 minutes at 37°C with a secondary antibody (1:200). In addition, Mito‐Tracker Green buffer (Beyotime) was incubated with cultured human NP cells for 40 minutes before fixation for mitochondrial localization. Protein expression was quantified based on the integrated optical density with the Image‐Pro Plus image analysis system.

### 2.9 Animal experiment

Two‐month‐old Sprague‐Dawley (SD) rats (200 g ± 20 g) were obtained from the Animal Experiment Center of Huazhong University of Science and Technology. Animal experimental protocols were approved by the Ethics Committee of Tongji Medical College, Huazhong University of Science and Technology. The SD rats were randomly divided into five groups of four. After the rats were anaesthetized with 10% chloral hydrate (0.3 mL/100 g), the injection site was disinfected with 1% active iodine, and lactate solutions (1 μL) of different concentrations (0 mM, 2 mM, 4 mM, 6 mM and 10 mM) were injected into the three IVDs closest to the root of the tail with 32G needles and microsyringes. After 6 weeks, MRI was used to detect the degree of disc degeneration, which was evaluated using the Pfirrmann grading system. After the MRI examination, the SD rats were sacrificed to obtain disc tissues for H&E staining.

A second experiment was conducted, in which SD rats were randomly divided into three groups of four. A solution (1 μL) containing amiloride (100 μM) and lactate (6 mM) was injected into rat tail discs. After 6 weeks, MRI was used to detect the degree of disc degeneration, which was evaluated using the Pfirrmann grading system. After the MRI examination, SD rats were sacrificed to obtain disc tissues for H&E and immunohistochemical staining for collagen II, aggrecan, ASIC1a, ASIC3, NLRP3, caspase‐1 and IL‐1β.

### 2.10 Statistical analysis

Data were analysed using GraphPad Prism 8 software and reported as the mean ± SD of the three independent experiments. Differences between group means were evaluated with Student's *t* test or one‐way ANOVA. Differences were considered statistically significant at *P* < .01.

## 3 RESULTS

### 3.1 Protein levels of ASIC1a, ASIC3 and NLRP3 inflammasome components are elevated in human IVDD tissue specimens

**FIGURE 1:**
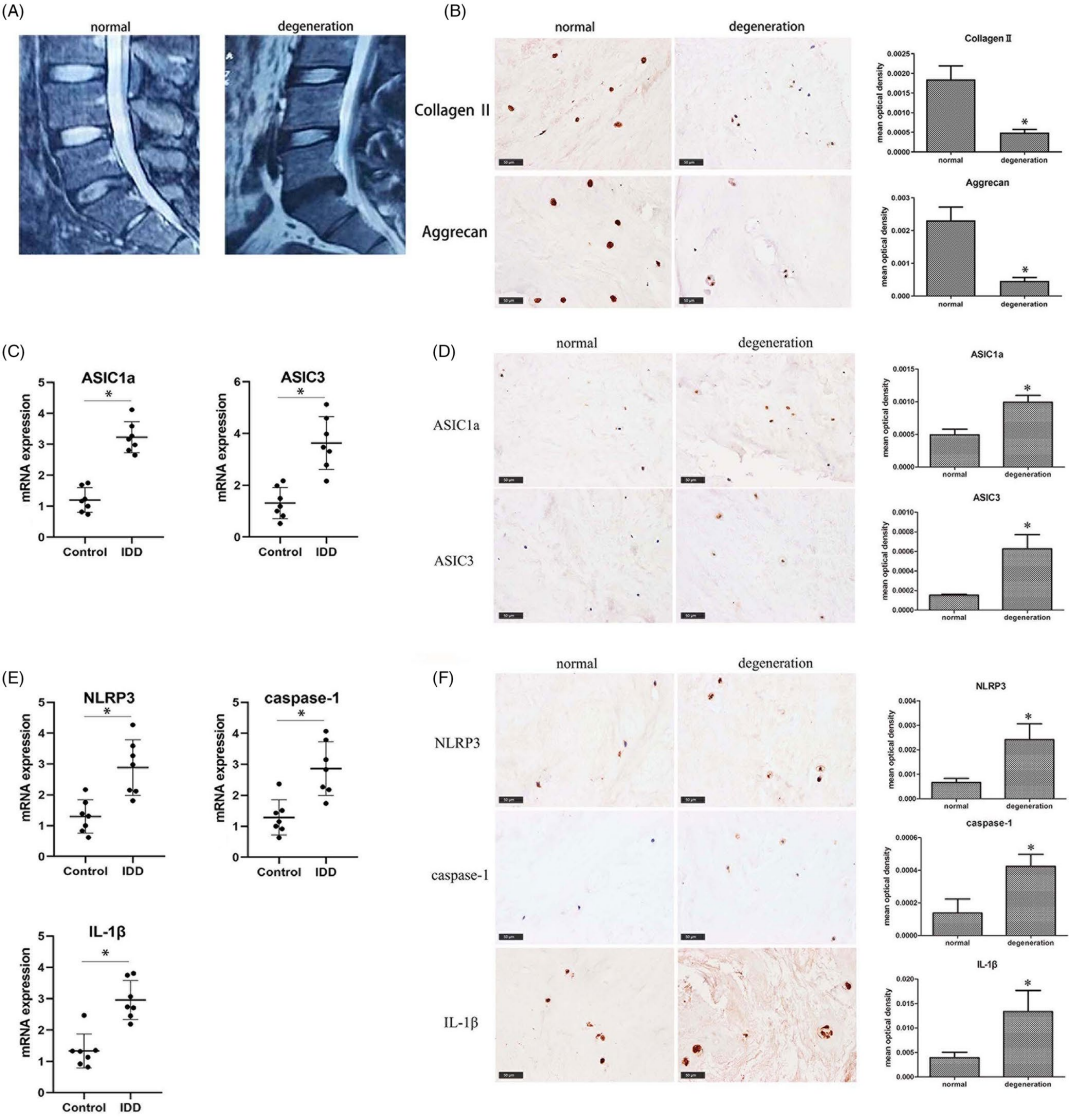
Expression Levels of ASIC1a/3 and NLRP3 Inflammasome Activation Were Higher in Degenerated Human NP Cells. (A) MRI images of normal and degenerated intervertebral discs. (B) Histochemical stain of a disc specimen; collagen Ⅱ and aggrecan contents were significantly lower in the IVDD specimen than in the normal specimen. Scale bar = 50 μm. (C) The mRNA expressions of ASIC1a and ASIC3 in normal and IVDD specimens were detected by RT‐PCR. (D) Histochemical stain of disc specimens; the ASIC1a and ASIC3 contents were significantly higher in the IVDD specimen than in the normal specimen. Scale bar = 50 μm. (E) The mRNA expression of NLRP3, CASP1 and IL‐1β in normal and IVDD specimens were detected by RT‐PCR. (F) Histochemical stain of disc specimens; the NLRP3, caspase‐1 and IL‐1β contents were significantly higher in the IVDD specimen than in the normal specimen. Scale bar = 50 μm. Data are represented as mean ± SD (n = 3). Significant differences between groups are indicated as * *P* < .01, compared with normal group

The degree of IVDD was graded based on MRI results according to the Pfirrmann grading system. We selected seven patients with three degrees or less of degeneration as a normal group and seven patients with four degrees or more of degeneration as a degeneration group. The MRI images from both these groups are shown in Figure 1A. The protein levels of collagen II and aggrecan were significantly lower in degenerative disc tissue samples than in normal tissue, as detected by immunohistochemical staining (Figure 1B). To study the role of ASICs in the process of disc degeneration, we first examined the mRNA expression and protein levels of ASICs and NLRP3 inflammasome components such as NLRP3, caspase‐1, and IL‐1β in normal and degenerated disc tissues. As shown in Figure 1, the mRNA expression of *ASIC1a* and *ASIC3* was substantially elevated in degenerative disc tissue samples relative to that in normal tissue (Figure 1C). The protein levels of ASIC1a and ASIC3 were significantly higher in degenerative disc tissue samples than in normal tissue, as revealed by immunohistochemical staining (Figure 1D). The mRNA expression of *NLRP3*, *CASP1* and *IL‐1β* was significantly increased in degenerative disc tissue samples relative to that in normal tissue, as shown by RT‐PCR (Figure 1E). Immunohistochemical staining showed that the protein levels of NLRP3, caspase‐1 and IL‐1β were significantly higher in degenerative disc tissue samples than in normal tissue (Figure 1F). There is therefore a positive correlation for ASIC1a, ASIC3 and NLRP3 with disc degeneration.

### 3.2 Lactate stimulates ASIC1a/ASIC3 expression and NLRP3 inflammasome activation in human NP cells

**FIGURE 2:**
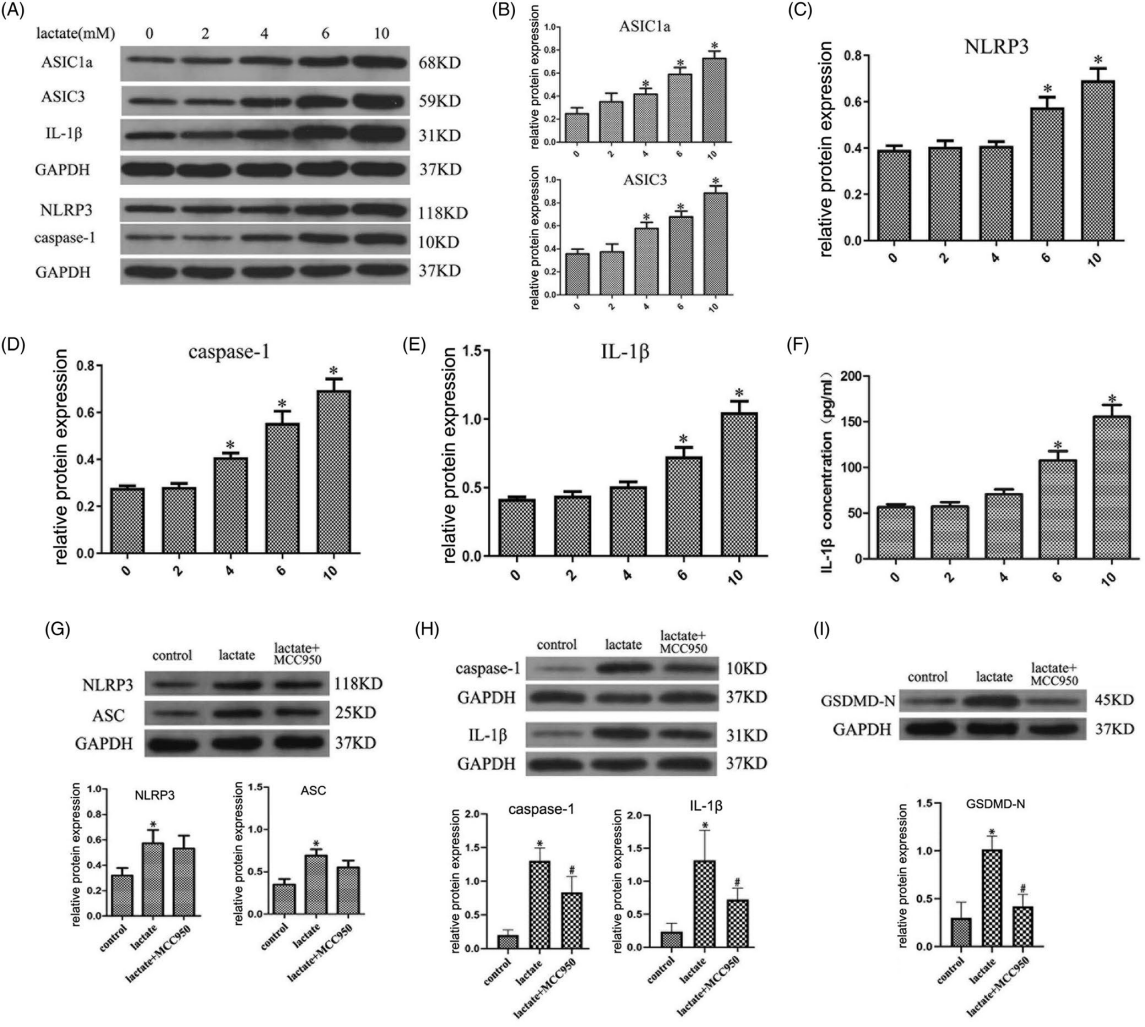
Lactate Stimulates ASIC1a/3 Expression and NLRP3 Inflammasome Activation in Human NP cells. Human NP cells were isolated from patients experiencing mild degeneration and stimulated with different concentrations of lactate for 24 h. MCC950 (10 μM) was used as a specific inhibitor to block NLRP3 inflammasome activation. (A) Western blot was used to detect the protein expression in human NP cells that were stimulated by different lactate concentrations (0‐10 mM). (B) Quantification of ASIC1a and ASIC3 immunoblots, (C) NLRP3 inflammasome‐related component immunoblots, (D) caspase‐1 immunoblots and (E) IL‐1β immunoblots. (F) The concentration of IL‐1β released to the extracellular matrix was examined by ELISA. (G) NLRP3 and ASC protein expressions were detected by immunoblot and quantification. (H) The caspase‐1 and IL‐1β contents in human nucleus pulposus cells were detected by immunoblot and quantification. (I) The GSDMD‐N content in human nucleus pulposus cells was detected by immunoblot and quantification. Data are represented as mean ± SD (n = 3). Significant differences between groups are indicated as *P < .01, compared with control group; ^#^ *P* < .01, compared with lactate group

To investigate the effects of ASIC1a and ASIC3 on NLRP3 inflammasome expression and activation in the degeneration process of human NP cells in vitro, we established a model of NP cell degeneration by stimulating human NP cells with different concentrations of lactate. We observed that dose‐dependent lactate substantially increased protein levels of ASIC1a, ASIC3, NLRP3, NLRP3 inflammasome component caspase‐1 and IL‐1β in NP cells in a dose‐dependent manner (Figure 2A‐E). ELISA revealed a significant increase in extracellular IL‐1β level upon lactate exposure (Figure 2F). To investigate the effect of NLRP3 inflammasome activation on NP pyroptosis, we used the NLRP3 inflammasome‐specific inhibitor MCC950 to inhibit its activation. As expected, 6 mM lactate increased NLRP3 and ASC protein expression, and MCC950 (10 μM) treatment did not block this process (Figure 2G). However, the expression of NLRP3 downstream proteins, such as caspase‐1, IL‐1β and GSDMD‐N, decreased with MCC950 treatment; levels of these proteins were otherwise elevated with lactate exposure (Figure 2H,I).

### 3.3 *Lactate promotes human NP cell pyroptosis* in vitro

**FIGURE 3:**
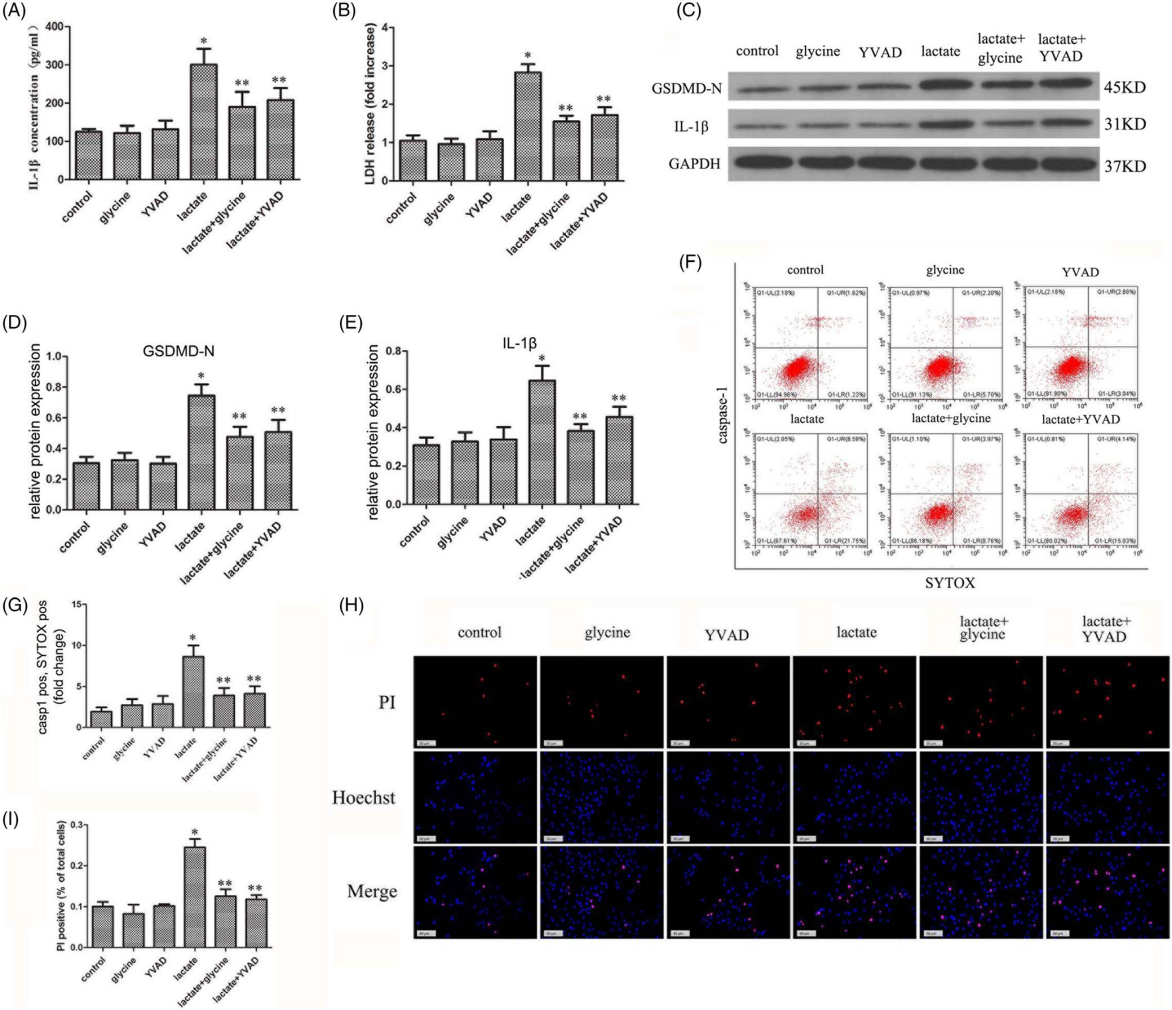
Lactate Increases Pyroptosis in Human NP Cells. (A) The lactate‐induced increase in IL‐1β release by human NP cells was detected by ELISA; this process was blocked by the pyroptosis inhibitors glycine and YVAD. (B) The increased LDH release by human NP cells was detected by ELISA; this process was also blocked by glycine and YVAD. (C‐E) Protein levels of GSDMD‐N and IL‐1β in human NP cells were examined by immunoblotting. (F, G) Pyroptosis level of human NP cells was examined by flow cytometry. (H, I) Immunofluorescence staining confirmed the death level of lactate‐stimulated human NP cells. Scale bar = 50 μm. Data are represented as mean ± SD (n = 3). Significant differences between groups are indicated as *P < .01, compared with control group; **P < .01, compared with lactate group

Pyroptosis is a cell death pattern associated with NLRP3 inflammasome activation. To study the effect of lactate on pyroptosis, we used the pyroptosis inhibitors glycine and YVAD to halt the pyroptosis process. Again, the levels of extracellular IL‐1β and LDH increased in the presence of 6 mM lactate but decreased in the presence of the pyroptosis inhibitors (Figure 3A,B). The levels of GSDMD‐N and IL‐1β in NP cells were increased in lactate‐treated groups but decreased in pyroptosis inhibitor groups (Figure 3C‐E). Pyroptosis levels of human NP cells showed a similar pattern to the Western blotting result when examined by flow cytometry and immunofluorescence staining (Figure 3F‐I). The pyroptosis level of NP cells was increased in the lactate (6 mM) treatment group, and this progress could be blocked by the pyroptosis inhibitors glycine and YVAD, as detected by flow cytometry (Figure 3F,G). Immunofluorescence staining showed the same result by using PI and Hoechst staining (Figure 3H,I). In conclusion, we demonstrated that extracellular lactate promotes pyroptosis in human NP cells in vitro.

### 3.4 NLRP3 inflammasome regulates the pyroptosis level of human NP cells stimulated by lactate

**FIGURE 4:**
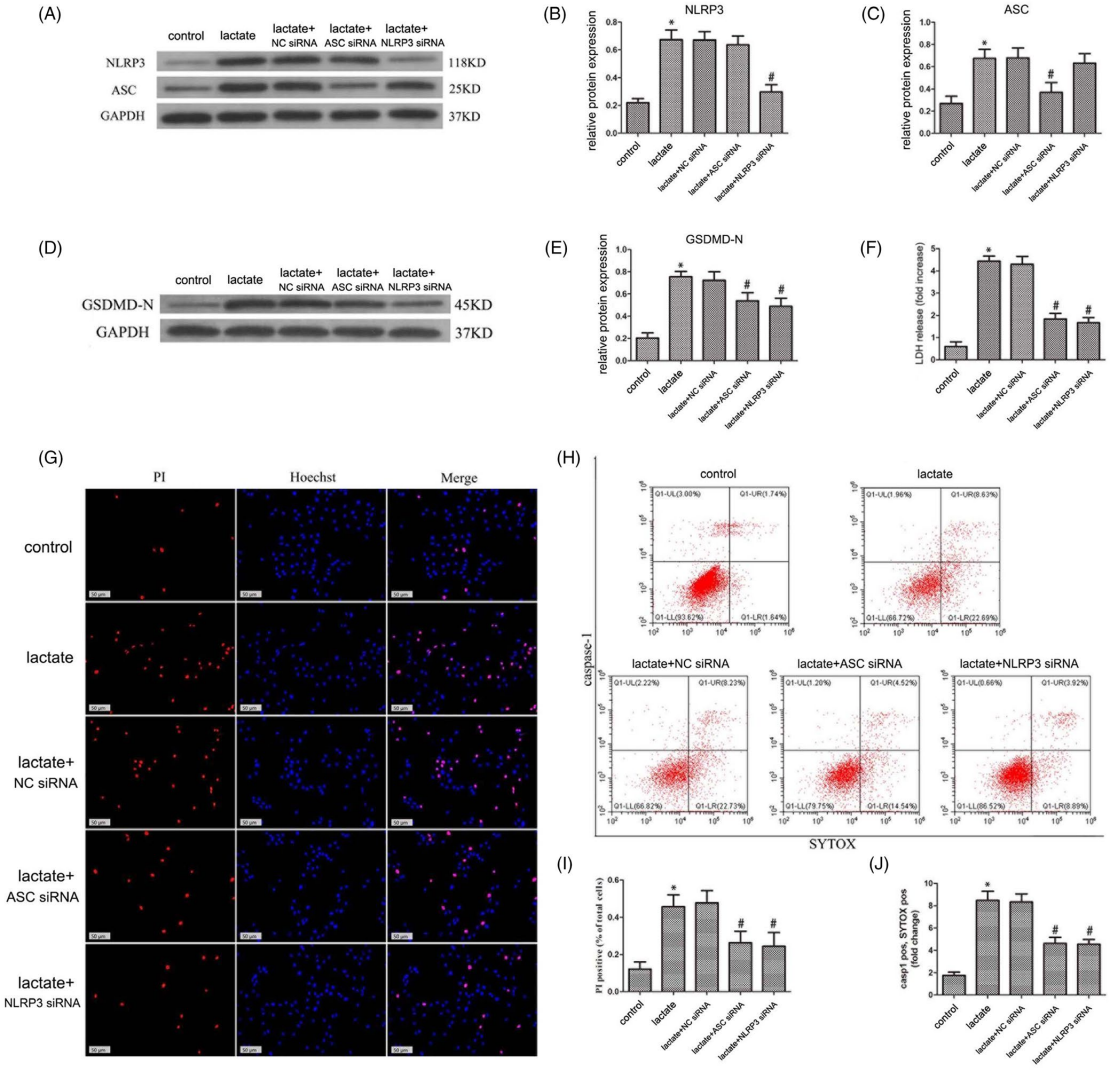
NLRP3 Promotes Pyroptosis of Human NP Cells Stimulated by Lactate. (A‐C) ASC and NLRP3 contents were decreased in human NP cells after transfection with ASC siRNA and NLRP3 siRNA, as confirmed by immunoblotting. (D, E) Protein level of GSDMD‐N in human NP cells was increased in the lactate group and decreased in the ASC siRNA and NLRP3 groups; levels were detected by immunoblotting. (F) LDH release by human NP cells was detected by flow cytometry. (G, I) The pyroptosis level of human NP cells was increased in the lactate group and decreased in the ASC siRNA and NLRP3 siRNA groups, as detected by immunofluorescence staining. Scale bar 50 = μm. (H, J) The pyroptosis level of human NP cells was increased in the lactate group and decreased in the ASC siRNA and NLRP3 siRNA groups, as detected by flow cytometry. Data are represented as mean ± SD (n = 3). Significant differences between groups are indicated as *P < .01, compared with control group; ^#^ *P* < .01, compared with lactate group

NLRP3 and ASC are important components of the NLRP3 inflammasome, essential for both assembly and activation. To study the role of NLRP3 in human NP cells stimulated by lactate, we constructed siRNAs against NLRP3 and ASC. RNAi using these siRNAs significantly decreased NLRP3 and ASC expression in human NP cells upon lactate stimulation (Figure 4A–C). Additionally, these siRNAs decreased the GSDMD‐N level in lactate‐stimulated NP cells (Figure 4D,E). The LDH level reduced in the NLRP3 and ASC siRNA‐treated groups (Figure 4F). The pyroptosis levels, as assessed by PI staining and flow cytometry, were significantly reduced in the NLRP3‐ and ASC siRNA‐treated groups (Figure 4G–J). These findings indicate that NLRP3 inflammasome activation regulates human NP cell pyroptosis.

### 3.5 NLRP3 inflammasome activation promotes lactate‐induced IVDD

**FIGURE 5:**
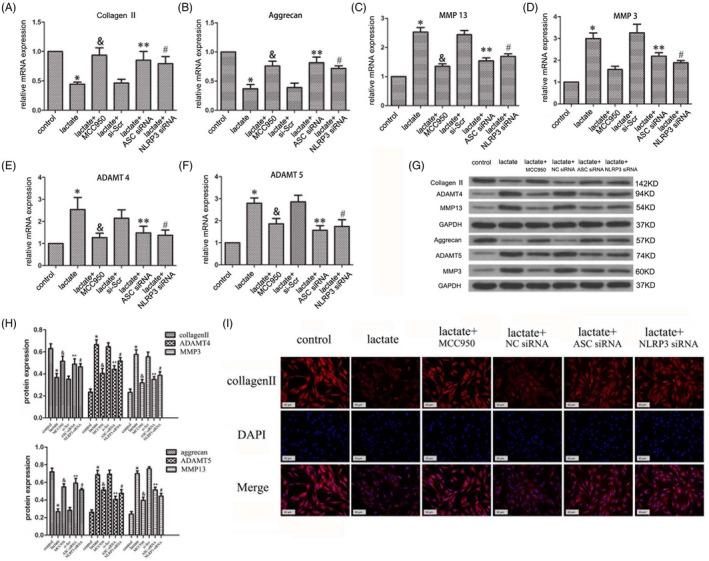
Lactate Promotes IVDD Via NLRP3 Inflammasome Activation. (A, B) Lactate decreases the mRNA expression of extracellular matrix components such as collagen Ⅱ and aggrecan; the inhibitor MCC950 (10 μM), ASC siRNA and NLRP3 siRNA blocked this process, as detected by RT‐PCR. (C‐F) Lactate decreased the mRNA expression of matrix proteases such as MMP3/13 and ADAMT4/5; the inhibitors MCC950 and ASC siRNA/NLRP3 siRNA blocked this process, as detected by RT‐PCR. (G, H) Lactate regulated the expression of extracellular matrix degeneration‐related proteins such as collagen Ⅱ, aggrecan, MMP3/13 and ADAMT4/5 in human NP cells, as detected by immunoblotting. (I) Immunofluorescence staining confirmed the degeneration of the extracellular matrix in lactate‐stimulated human NP cells. Scale bar = 50 μm. Data are represented as mean ± SD (n = 3). Significant differences between groups are indicated as *P < .01, compared with control group; **P < .01, compared with lactate group; ^#^ *P* < .01, compared with lactate group; and *P* < .01, compared with lactate group

Next, we investigated whether NLRP3 plays a role in the progression of IVDD using the NP cell model. The mRNA expression of the ECM proteins collagen II and aggrecan decreased in the lactate‐treated groups and increased in the MCC950‐, ASC siRNA‐ and NLRP3 siRNA‐treated groups (Figure 5A,B). There were concomitant increases in the mRNA levels of the ECM degradation‐associated proteases MMP3, MMP13, ADAMT4 and ADAMT5 in the lactate‐treated groups; however, their levels decreased in the MCC950‐ and siRNA‐treated groups (Figure 5C–F). The levels of ECM proteins and ECM degeneration‐associated proteases were then examined by immunoblotting. Consistent with the mRNA results, lactate decreased the levels of collagen II and aggrecan and increased the levels of ECM degradation‐associated proteases (Figure 5G–H), whereas MCC950 and siRNAs against NLRP3 and ASC had the opposite effect. Again, immunofluorescence results confirmed these findings (Figure 5I). Therefore, lactate‐induced NLRP3 inflammasome activation promotes the degeneration of the ECM of human NP cells.

### 3.6 ASIC1a and ASIC3 play important roles in NLRP3 inflammasome activation and lactate‐induced pyroptosis of NP cells

**FIGURE 6:**
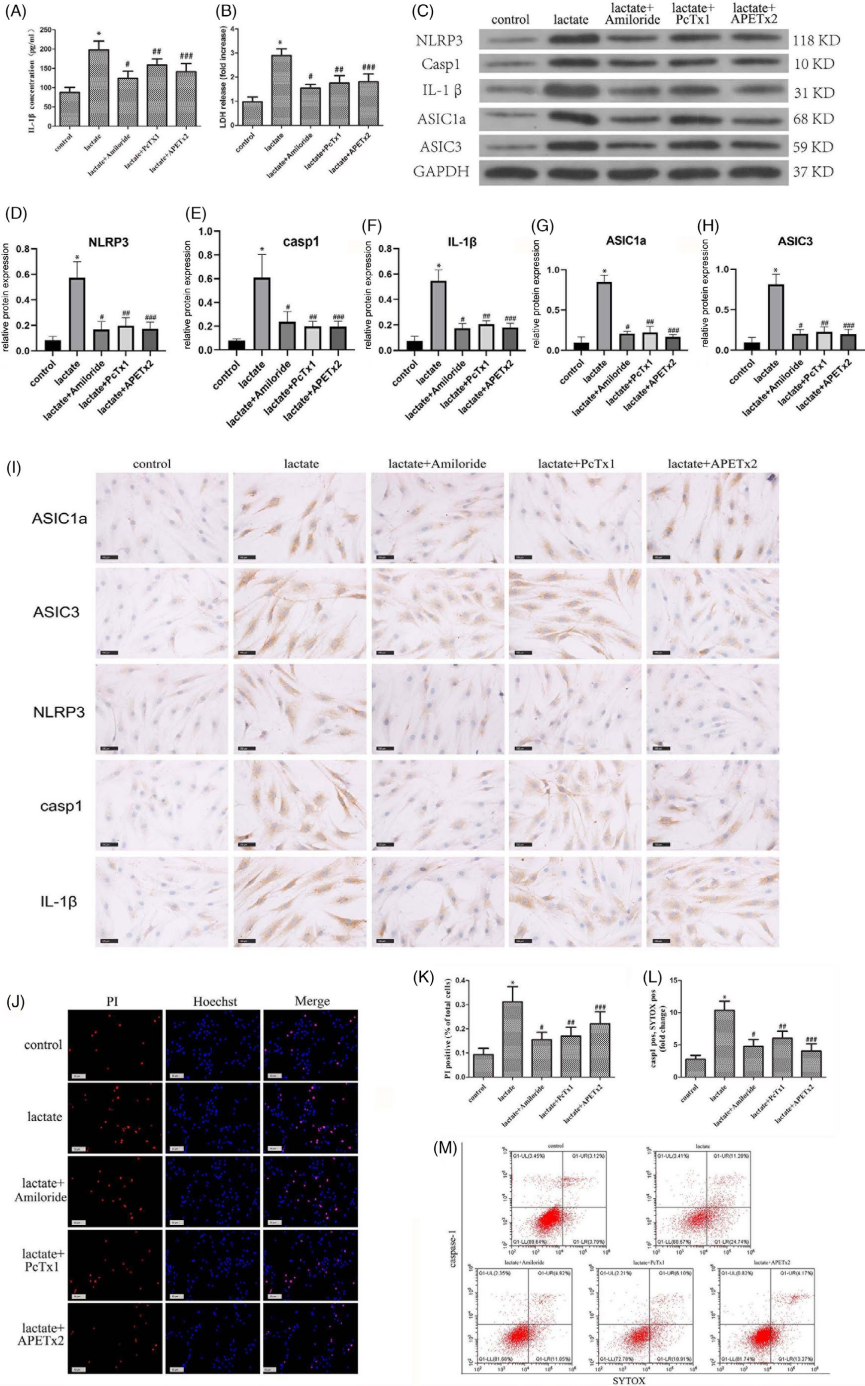
ASIC1a and ASIC3 Play Important Roles in Lactate‐induced NLRP3 Inflammasome Activation and Pyroptosis of Nucleus Pulposus Cells. Human NP cells were stimulated with lactate for 24 h. Amiloride (ASIC inhibitor, 100 μM), PcTx1 (ASIC1a inhibitor, 20 nM) and APETx2 (ASIC3 inhibitor, 2 μm) were used to inhibit the function of ASICs, including ASIC1a and ASIC3. (A) Extracellular IL‐1β release level and (B) extracellular LDH release level in human NP cells were detected by ELISA. (C) Protein levels of NLRP3, caspase‐1, IL‐1β, ASIC1a and ASIC3 in human NP cells examined by immunoblotting. Quantification of (D) NLRP3 immunoblots, (E) caspase‐1 immunoblots, (F) IL‐1β immunoblotting, (G) ASIC1a immunoblots and (H) ASIC3 immunoblots. (I) Protein levels of NLRP3, caspase‐1, IL‐1β, ASIC1a, ASIC3 in human NP cells were detected by immunohistochemical staining. Scale bar = 100 μm. (J, K) Pyroptosis level of human NP cells detected by immunofluorescence staining. Scale bar = 50 μm. (L, M) Pyroptosis level of human NP cells detected by flow cytometry. Data represent mean ± SD (n = 3). Significant differences between groups are indicated as *P < .01, compared with control group; ^#^ *P* < .01, compared with lactate group; ^##^ *P* < .01, compared with lactate group; ^###^ *P* < .01, compared with lactate group

To delineate the roles of ASIC1a and ASIC3 in NLRP3 inflammasome activation and pyroptosis in NP cells stimulated by lactate, we used the ASIC inhibitors amiloride, PcTx1 and APETx2. Amiloride inhibits both ASIC1a and ASIC3, while PcTx1 specifically inhibits ASIC1a, and APETx2 specifically inhibits ASIC3. All three inhibitors decreased the levels of extracellular IL‐1β and LDH (Figure 6A,B). Immunoblotting demonstrated that the levels of NLRP3 inflammasome components were decreased upon ASIC inhibition (Figure 6C–H). This was further confirmed by immunohistochemical staining (Figure 6I). Immunofluorescence and flow cytometry showed that pyroptosis levels of human NP cells were lower in the groups that were treated with amiloride, PcTx1 or APETx2 (Figure 6J–M). Thus, we conclude that ASIC1a and ASIC3 are necessary for NLRP3 inflammasome component expression and activation in human NP cells.

### 3.7 Reactive oxygen species (ROS) regulate NLRP3 inflammasome activation and pyroptosis via the NF‐κB signalling pathway in lactate‐stimulated human NP cells

**FIGURE 7:**
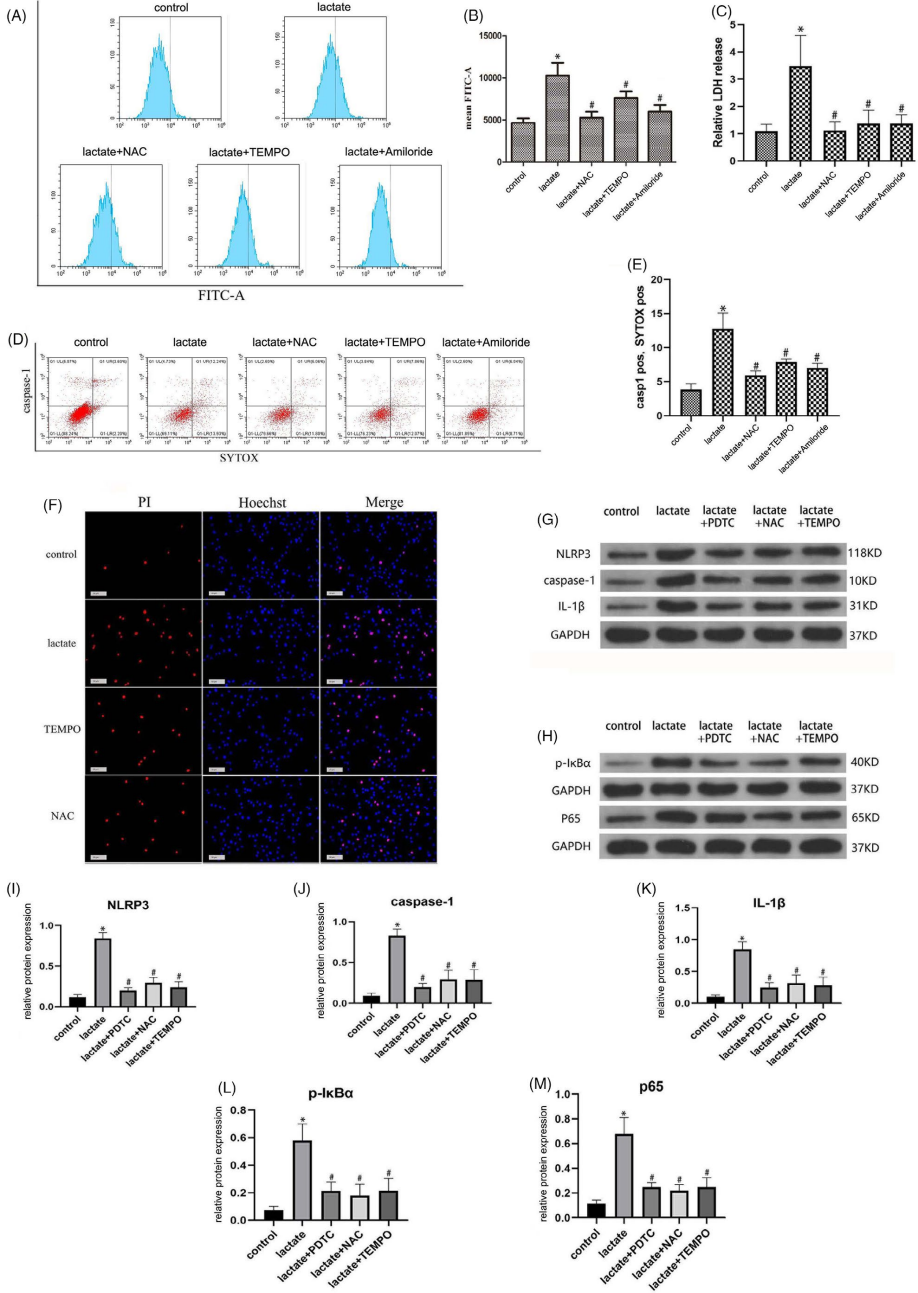
ASIC1a and ASIC3 Regulate NLRP3 Inflammasome Activation and Pyroptosis through the ROS/NF‐κB signalling pathway in lactate‐stimulated Human NP Cells. ROS scavengers TEMPO (50 μM) and NAC (10 mM) were used to reduce ROS levels in human NP cells. ASICs inhibitor Amiloride (100 μM) was used to block ASICs channel fuction.(A) ROS level of human NP cells was detected by flow cytometry. (B) Quantification of ROS level. (C) LDH release levels in human NP cells detected by ELISA. (D) Pyroptosis level of human NP cells examined by flow cytometry. (E) Quantification of pyroptosis level detected by flow cytometry. (F) Pyroptosis level of human NP cells examined by immunofluorescence staining. Scale bar = 50 μm. (G) Protein levels of NLRP3, caspase‐1 and IL‐1β in human NP cells were detected by immunoblot. (H) Protein levels of p‐IκBα and p65 in human NP cells were detected by immunoblot. Quantification of (I) NLRP3 immunoblot, (J) caspase‐1 immunoblots, (K) IL‐1β immunoblot, (L) p‐IκBα immunoblots and (M) p65 immunoblots. Data are represented as mean ± SD (n = 3). Significant differences between groups are indicated as *P < .01, compared with control group; ^#^ *P* < .01, compared with lactate group

To investigate the mechanism by which ASIC1a and ASIC3 promote NLRP3 inflammasome activation, we examined the intracellular ROS in human NP cells upon lactate stimulation. The ROS scavengers NAC (10 mM) and TEMPO (50 μm) were used to reduce the intracellular ROS level. Both scavengers decreased intracellular ROS (Figure 7A,B) and extracellular LDH levels (Figure 7C). In addition, immunofluorescence and flow cytometry revealed that the pyroptosis level was reduced by the ROS scavengers (Figure 7D–F). To confirm that the NF‐κB signalling pathway participated in this regulation process, we used the NF‐κB inhibitor PDTC (100 μM) and examined the NF‐κB‐associated components p‐IκBa and p65, which are metabolites involved in NF‐κB activation and the increased expression of which indicates the activation of this pathway. PDTC significantly blocked the NF‐κB signalling pathway and inhibited NLRP3 inflammasome expression activation (Figure 7G–M). Similarly, the ROS scavengers NAC and TEMPO blocked the NF‐κB signalling pathway (Figure 7H,L,M).

### 3.8 *Lactate regulates the IVDD process via ASCI1a/ASIC3 and NLRP3 inflammasome activation* in vivo

**FIGURE 8:**
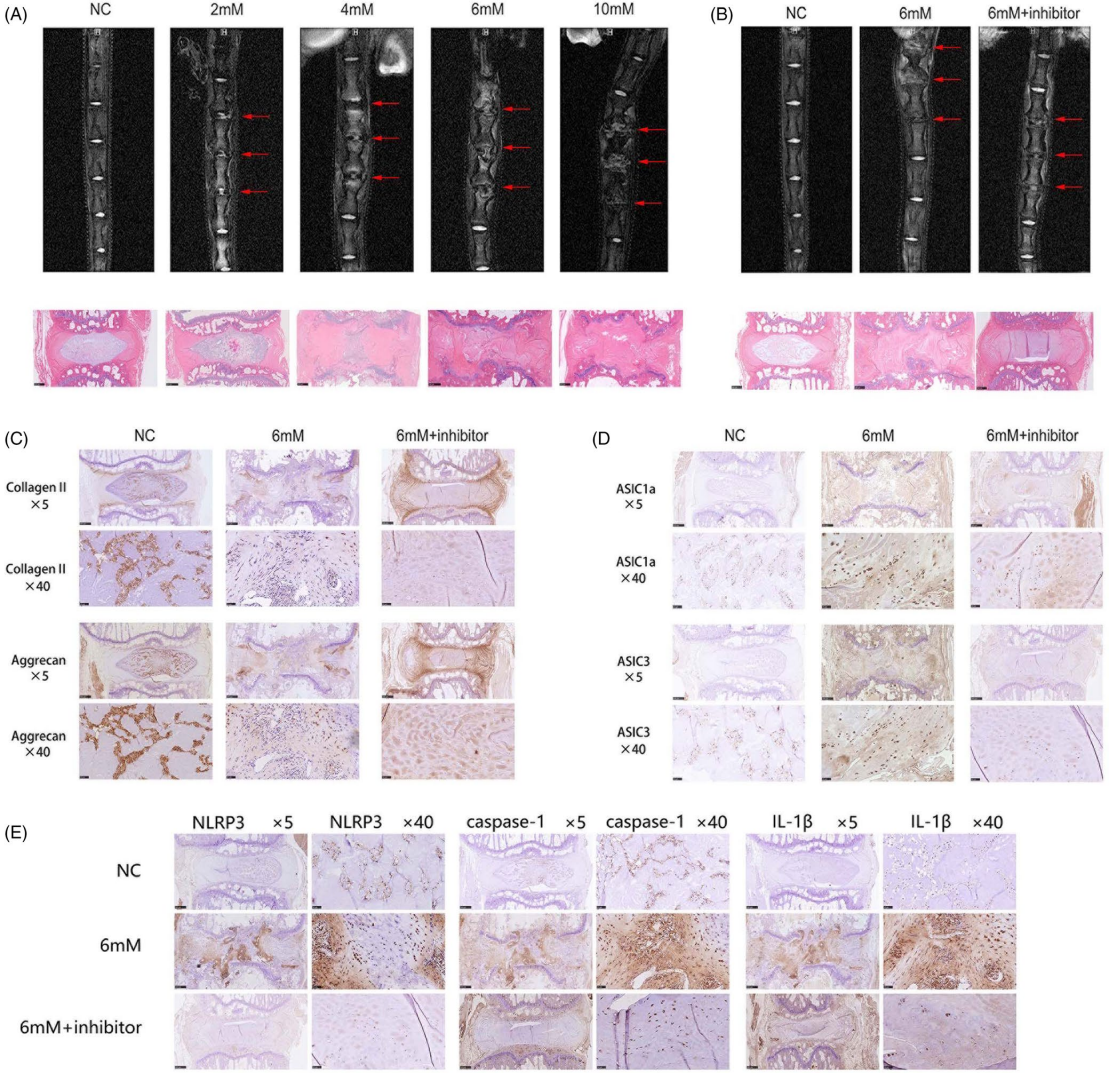
ASIC1a and ASIC3 Relieve Lactate‐induced Intervertebral Disc Degeneration in Vivo. (A) The degeneration of rat tail discs stimulated by different lactate concentration was detected by MRI and immunohistochemical staining. (B) The degeneration of rat tail discs stimulated by lactate and ASIC inhibitor (amiloride, 100 μL) was detected by MRI and immunohistochemical staining. (C) Protein levels of extracellular matrix components collagen Ⅱ and aggrecan in human NP cells; ×5 scale bar = 50 μm; ×40 scale bar = 500 μm. (D) Protein levels of ASIC1a and ASIC3 in human NP cells. ×5 scale bar = 50 μm; ×40 scale bar = 500 μm. (E) Protein levels of NLRP3 inflammasome components, caspase‐1 and IL‐1β in human NP cells. ×5 scale bar = 50 μm; ×40 scale bar = 500 μm. Data are represented as mean ± SD (n = 3)

To study the effect of lactate on disc degeneration in vivo, we used a microsyringe to inject lactate into the rat tail disc tissue. MRI and H&E staining used to examine the level of IVD degeneration demonstrated that lactate strongly stimulated the degeneration of the IVD tissue in a dose‐dependent manner (Figure 8A). To delineate the roles of ASIC1a and ASIC3 in lactate‐stimulated disc degeneration in vivo, the ASIC inhibitor amiloride was used. Amiloride significantly mitigated disc degeneration caused by lactate (Figure 8B). Next, we examined the effects of lactate on the morphology of discs. As shown in Figure 8C, lactate inhibited the expression of collagen Ⅱ and aggrecan, and amiloride inhibited these changes. Immunohistochemical staining for ASIC1a, ASIC3 and NLRP3 inflammasome components in rat tail discs demonstrated that lactate increased the expression levels of all three, while amiloride reduced their expression (Figure 8D,E).

## 4 DISCUSSION

Here, we report the crucial roles of ASIC1a and ASIC3 in driving NLRP3 inflammasome activation and pyroptosis in lactate‐stimulated NP cells. ASICs are expressed in IVD cells and promote the expression of MMPs and ADAMTs, which induce ECM degeneration.^30^, ^34^ However, the mechanism by which this occurs remains poorly understood till date. We show here that ASIC1a and ASIC3 induce NLRP3 inflammasome activation and pyroptosis in NP cells through the ROS/NF‐κB signalling pathway.

High lactate concentration is a pathogenic factor for IVDD and has been reported to regulate NP cell apoptosis, autophagy and matrix synthesis.^14^ IVDD is characterized by high levels of proinflammatory cytokines such as TNF‐α and IL‐1β, which are secreted by IVD cells.^35^ In addition, extracellular acidosis has been reported to trigger NLRP3 inflammasome activation and IL‐1β release in the innate immune response.^36^ However, it was unclear whether high lactate concentrations induce inflammation in NP cells. Here, we found that the levels of NLRP3 inflammasome activation, IL‐1β release and pyroptosis increased significantly under high extracellular lactate concentrations. IL‐1β is an important inflammatory cytokine that has been reported to be closely linked to disc degeneration.^32^, ^37^ Previous studies have demonstrated that IL‐1β is expressed at significantly higher levels in painful discs than in pain‐free discs and that the expression of IL‐1β increases at higher levels of IVDD.^38^ Therefore, IVD tissue would be susceptible to degeneration if the negative regulation of the IL‐1β signal was lacking.^39^

Inflammation is regarded as a critical regulator of IVDD. NP cells, the main cellular components of IVD tissue, play important roles in IVD inflammation. However, the signalling pathway by which extracellular lactate regulates the expression and release of inflammatory factors was previously unclear. ASICs are ion channels that activate signal pathways and regulate gene expression when activated by extracellular acid, which stimulates human monocytes to produce IL‐1β and thus induces inflammation.^40^, ^41^ ASIC1a and ASIC3 are expressed in innate immune cells such as macrophages and dendritic cells.^42^, ^43^ Sluka and Gregory demonstrated that ASICs play a clear role in inflammatory muscle and joint pain.^44^ In this study, we found that there was a significant increase in ASIC1a and ASIC3 in the NP of degenerated IVDs and that ASIC1a and ASIC3 regulate the degeneration of the ECM via NF‐κB phosphorylation.

Extracellular acidosis has been reported to trigger NLRP3 inflammasome activation and IL‐1β release as part of the innate immune response. Here, we found that ASIC1a and ASIC3 expression levels are positively correlated with NLRP3 inflammasome activation and IL‐1β release. ASICs regulate transmembrane Ca^2+^ influx in response to extracellular acidification.^25^ Ca^2+^ influx is a critical second messenger in signal transduction pathways that modulate diverse physiological functions. Increased intracellular Ca^2+^ signalling has been associated with increase in ROS and NLRP3 inflammasome activation in human peripheral blood mononuclear cells.^45^ We found that intercellular ROS level decreased if NP cells were stimulated by lactate disposed by the Ca^2+^ inhibitor CPA (30 μM) (Figure 7). Thus, the ROS level was regulated by Ca^2+^ influx. Then, we found that NLRP3 inflammasome activation was decreased when NP cells were stimulated by lactate disposed by the ROS scavengers NAC and TEMPO. We therefore concluded that ASIC1a and ASIC3 contributed to the inflammation of NP cells via ROS production when NP cells were stimulated by extracellular lactate. We found that ASIC1a and ASIC3 are activated by extracellular lactic acid, subsequently inducing an increase in ROS levels in NP cells. We further found that NLRP3 inflammasome activation and IL‐1β release were reduced when intercellular ROS were eliminated. The ion fluxion stimulated an increase in ROS that could activate the NF‐κB signalling pathway and thereby promote *NLRP3* expression. Moreover, NLRP3 activation and IL‐1β release were reduced when intracellular ROS were absent or the NF‐κB signalling pathway was inhibited.

**FIGURE 9:**
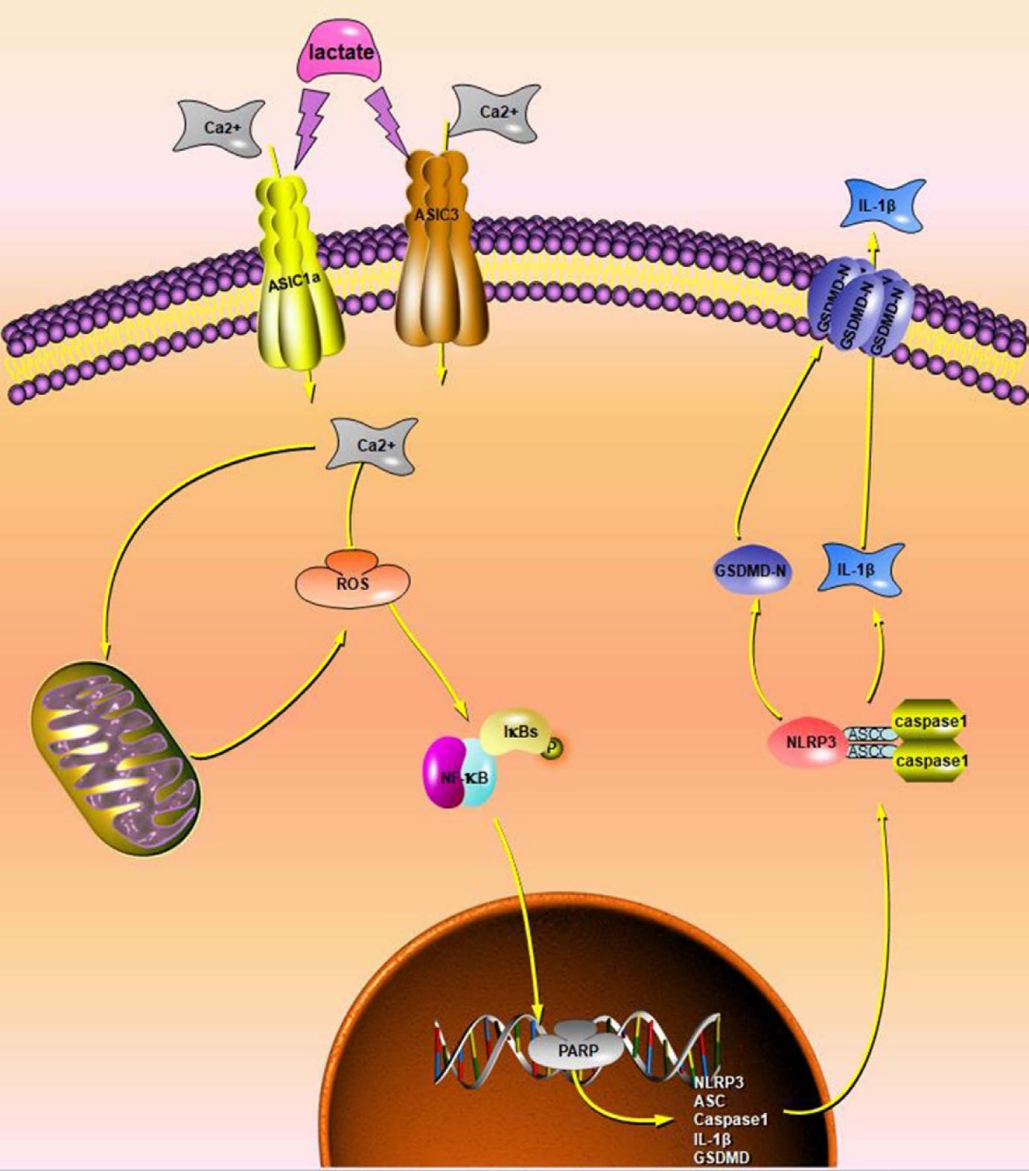
Schematic Illustration of NLRP3 Inflammasome Activation and Pyroptosis in Lactate‐stimulated Human NP Cells. Extracellular lactate induces Ca^2+^ influx by stimulating ASIC1a and ASIC3 on the cell membrane. As a second messenger, Ca^2+^ promotes an increase in intracellular ROS. ROS increases NLRP3 inflammasome component expression through the NF‐κB signalling pathway. NLRP3 components assemble and activate in the cells, leading to IL‐1β release and pyroptosis

Inflammasomes are inflammation‐inducing protein complexes that widely occur in many kinds of cells. NLRP3, which is currently the most studied inflammasome, assembles with ASC and pro‐caspase‐1 when the cell is stimulated by PAMPs or DAMPs. The mature caspase‐1 then promotes IL‐1β production and release. We found that NLRP3 expression was regulated by ASIC1a and ASIC3 in lactate‐stimulated NP cells. Recent studies have described pyroptosis, a newly discovered programmed cell death, which accompanies IL‐1β release. As it has been demonstrated that GSDMD‐N is necessary and sufficient for pyroptosis,^46^, ^47^ the expression of GSDMD‐N could be considered a marker of pyroptosis. We found a high level of GSDMD‐N expression to be associated with death in lactate‐stimulated NP cells. In addition, we found that GSDMD‐N expression and NP cell pyroptosis decreased when NLRP3 inflammasome or caspase‐1 activation was inhibited. We have therefore demonstrated that ASIC1a and ASIC3 are important ion channels in the cell membrane. They are influenced by changes in extracellular lactate levels, inducing influx of Ca^2+^, which as a critical second messenger in signal transduction pathways, promotes the level of intercellular ROS and then induces NF‐κB signalling pathway activation. The NF‐κB signalling pathway is an inflammation‐activating signalling pathway, and it can promote the expression of NLRP3 inflammasome components. The NLRP3 inflammasome is activated when its components assemble. The activation of the NLRP3 inflammasome promotes the maturation and release of the inflammatory factor IL‐1β (Figure 9).

To verify the role of lactate in IVDD in vivo, we used a rat model and injected lactate into the rat tail disc. The MRI and H&E staining results show that lactate can significantly induce IVDD, while the immunohistochemical staining results demonstrate that ASIC1a, ASIC3 and the NLRP3 inflammasome participate in this process. Groups that were pretreated with ASIC antagonists showed a lower level of IVDD decrease than the groups that were treated with only lactate. This demonstrates that ASICs promote the progression of IVDD.

We conclude that ASIC1a and ASIC3 play important roles in IVDD progression by regulating NLRP3 inflammasome activation and pyroptosis, which induce ECM degeneration and cell death in lactate‐stimulated NP cells. ASIC1a and ASIC3 further promote IVDD progression by modulating NLRP3 inflammasome component expression through the NF‐κB signalling pathway. Therefore, ASICs hold promise as potential targets for the treatment of various diseases in the future.

## CONFLICT OF INTEREST

The authors declare that they have no conflict of interest.

## AUTHOR CONTRIBUTIONS

Cao Yang, Kangcheng Zhao, Ran An and Qian Xiang conceived and designed the study. Kangcheng Zhao, Ran An, Qian Xiang, Gaocai Li, Kun Wang, Yu Song and Zhiwei Liao acquired, analysed and interpreted the data. Kangcheng Zhao, Cao Yang, Ran An, Shuai Li, Xiaobo Feng, and Wenbin Hua drafted and edited the manuscript. Kangcheng Zhao, Cao Yang, Ran An, Xinghuo Wu, Yukun Zhang, Abhirup Das, Gaocai Li, Kun Wang, Shuai Li, Xiaobo Feng and Wenbin Hua critically revised the manuscript for intellectual content. All authors approved the final version of the manuscript.

## Data Availability

The data that support the findings of this study are available from the corresponding author upon reasonable request.
